# CT Body Composition of Sarcopenia and Sarcopenic Obesity: Predictors of Postoperative Complications and Survival in Patients with Locally Advanced Esophageal Adenocarcinoma

**DOI:** 10.3390/cancers13122921

**Published:** 2021-06-11

**Authors:** Uli Fehrenbach, Tilo Wuensch, Pia Gabriel, Laura Segger, Takeru Yamaguchi, Timo Alexander Auer, Nick Lasse Beetz, Christian Denecke, Dino Kröll, Jonas Raakow, Sebastian Knitter, Sascha Chopra, Peter Thuss-Patience, Johann Pratschke, Bernd Hamm, Matthias Biebl, Dominik Geisel

**Affiliations:** 1 Department of Radiology, Charité-Universitätsmedizin Berlin, 13353 Berlin, Germany; pia.gabriel@charite.de (P.G.); Laura.segger@charite.de (L.S.); timo-alexander.auer@charite.de (T.A.A.); nick-lasse.beetz@charite.de (N.L.B.); Bernd.Hamm@charite.de (B.H.); dominik.geisel@charite.de (D.G.); 2 Department of Surgery, Campus Charité Mitte and Campus Virchow Klinikum, Charité-Universitätsmedizin Berlin, 13353 Berlin, Germany; tilo.wuensch@charite.de (T.W.); christian.denecke@charite.de (C.D.); dino.kroell@charite.de (D.K.); jonas.raakow@charite.de (J.R.); sebastian.knitter@charite.de (S.K.); sascha.chopra@charite.de (S.C.); johann.pratschke@charite.de (J.P.); matthias.biebl@charite.de (M.B.); 3 Department of Radiology, Kobe University Graduate School of Medicine, Kobe 6500017, Japan; takeru.yamaguchi1216@gmail.com; 4 Berlin Institute of Health (BIH), 10178 Berlin, Germany; 5 Department of Hematology, Oncology and Cancer Immunology, Charité-Universitätsmedizin Berlin, 13353 Berlin, Germany; peter.thuss@charite.de

**Keywords:** computed tomography, body composition, sarcopenia, sarcopenic obesity, esophageal cancer, surgery

## Abstract

**Simple Summary:**

The incidence of esophageal cancer is constantly rising and patients are often diagnosed at an advanced stage. Surgical resection, if possible, is the curative treatment of choice. However, esophagectomy for cancer is a major surgical procedure and is associated with perioperative morbidity. Preoperative staging examinations are carried out on every patient, and imaging datasets contain valuable information about the patient’s physical condition beyond the routinely assessed tumor extent. In this study, the abdominal muscle and fat mass were quantified during the preoperative staging and postoperative follow-up of 85 patients with locally advanced esophageal adenocarcinoma, and these imaging biomarkers were correlated with surgical complications and patient outcomes. Our analysis showed that sarcopenic patients with low muscle mass were more likely to have major complications and that hospitalization was prolonged, especially in patients with sarcopenic obesity. Low preoperative muscle mass and its decrease during the follow-up also predicted poorer overall survival.

**Abstract:**

Background: To assess the impact of body composition imaging biomarkers in computed tomography (CT) on the perioperative morbidity and survival after surgery of patients with esophageal cancer (EC). Methods: Eighty-five patients who underwent esophagectomy for locally advanced EC after neoadjuvant therapy between 2014 and 2019 were retrospectively enrolled. Pre- and postoperative CT scans were used to assess the body composition imaging biomarkers (visceral (VAT) and subcutaneous adipose tissue (SAT) areas, psoas muscle area (PMA) and volume (PMV), total abdominal muscle area (TAMA)). Sarcopenia was defined as lumbar skeletal muscle index (LSMI) ≤38.5 cm^2^/m^2^ in women and ≤52.4 cm^2^/m^2^ in men. Patients with a body mass index (BMI) of ≥30 were considered obese. These imaging biomarkers were correlated with major complications, anastomotic leakage, postoperative pneumonia, duration of postoperative hospitalization, disease-free survival (DFS), and overall survival (OS). Results: Preoperatively, sarcopenia was identified in 58 patients (68.2%), and sarcopenic obesity was present in 7 patients (8.2%). Sarcopenic patients were found to have an elevated risk for the occurrence of major complications (OR: 2.587, *p* = 0.048) and prolonged hospitalization (32 d vs. 19 d, *p* = 0.040). Patients with sarcopenic obesity had a significantly higher risk for postoperative pneumonia (OR: 6.364 *p* = 0.018) and a longer postoperative hospital stay (71 d vs. 24 d, *p* = 0.021). Neither sarcopenia nor sarcopenic obesity was an independent risk factor for the occurrence of anastomotic leakage (*p* > 0.05). Low preoperative muscle biomarkers (PMA and PMV) and their decrease (ΔPMV and ΔTAMA) during the follow-up period significantly correlated with shorter DFS and OS (*p* = 0.005 to 0.048). Conclusion: CT body composition imaging biomarkers can identify high-risk patients with locally advanced esophageal cancer undergoing surgery. Sarcopenic patients have a higher risk of major complications, and patients with sarcopenic obesity are more prone to postoperative pneumonia. Sarcopenia and sarcopenic obesity are both subsequently associated with a prolonged hospitalization. Low preoperative muscle mass and its decrease during the postoperative follow-up are associated with lower DFS and OS.

## 1. Introduction

Esophageal cancer (EC) is the eighth most common cancer globally, occurs more frequently in men, and has an unfavorable prognosis with the sixth highest mortality rate [1,2,3]. The overall incidence of EC has constantly been rising over the past decades as many associated habits have been on the rise in the general population [4,5]. Symptoms occur late, and patients typically have advanced EC at the time of diagnosis [6]. If general operability is given, surgical resection remains the best curative treatment option after neoadjuvant treatment for locally advanced cancers [7,8,9,10]. However, careful patient selection with an upfront assessment of the operative risk is necessary to improve surgical outcomes [11]. The most common surgical technique is a total minimally invasive esophagectomy or open esophagogastrostomy [8]. Surgical removal of EC is an extensive operation with a range of peri- and postoperative complications, including anastomotic leakage, bleeding, and postoperative pneumonia [12,13,14,15].

Assessment of body composition based on computed tomography (CT) has been evaluated in various groups of patients to assess the possible effects of sarcopenia on patient outcome. Research in the field of body composition was initially focused primarily on patients with cardiovascular diseases, but soon shifted to cancer patients [16]. In cancer patients, sarcopenia has moved into the spotlight over recent decades, and several studies have shown that pretherapeutic sarcopenia is associated with poor outcomes after subsequent cancer treatment [17]. There have been several attempts to assess sarcopenia in cancer patients using a variety of conventional methods such as BMI, waist-to hip-ratio, bio-impedance analysis (BIA), and imaging-based techniques like dual-energy X-ray absorptiometry, MRI, and CT [17,18,19,20,21]. CT-based assessment of sarcopenia commonly relies on quantification of the skeletal muscle mass [18].

Although every patient undergoes CT imaging as part of staging prior to surgery of esophageal cancer, assessment of the CT dataset is mostly limited to direct, cancer-related aspects such as tumor extent and presence of metastases [22]. The valuable information CT images provide on body composition and potential predictors of patient fitness has not been used in routine clinical practice.

Thus, the purpose of this study was to evaluate CT body composition imaging biomarkers as potential predictors of perioperative morbidity and postoperative outcome in patients undergoing surgery for locally advanced EC.

## 2. Material and Methods

### 2.1. Patients and Clinical Data

The Department of Surgery’s database was searched for patients with locally advanced esophageal adenocarcinoma who underwent oncological esophagectomy between January 2014 and January 2019, with CT imaging data available as indicated below. A total of 85 consecutive patients were found to be eligible to be included in this retrospective study. The following clinical data of these patients were retrieved: basic patient information (age, sex, body weight and height), surgical technique (open, laparoscopic, hybrid or robotic), preoperative chemotherapy +/− radiation therapy (yes/no), UICC stage, postoperative complications (overall, major complications, anastomotic leakage, pneumonia), duration of postoperative hospitalization, disease-free survival and overall survival.

Minimally invasive Ivor Lewis resection comprised total minimal invasive operations, hybrid procedures (with one part of the operation being performed as an open procedure), and total minimally invasive robotic resections through an abdominal and right-thoracic approach. Our standard minimally invasive approach was the total minimally invasive procedure and reasons for a hybrid approach were additional abdominal organ resection, status post extensive previous abdominal surgery, demand for D3 lymphadenectomy or extensive intra-abdominal adhesions (for abdominal open procedures), or extensive pleural adhesions, suspected T4 situations (except for concomitant VATS lung resections) or bulky lymphatic involvement at the tracheal bifurcation (for open thoracic procedures). Despite the difference in approaches, the surgical technique was standardized between groups and performed as described previously [23,24]. All operations were performed by two consultant surgeons. A major intraoperative and postoperative complication was defined as a surgical or medical complication with a Clavien−Dindo grade of II or higher [25].

Exclusion criteria were insufficient clinical data, postoperative histology other than esophageal adenocarcinoma, and patient age <18 years.

### 2.2. Imaging

Preoperative CT scans, those obtained after completion of neoadjuvant therapy and before surgery were used for analysis. Follow-up CT scans of the patients were selected postoperatively over a period of two months to two years. If several examinations were available during this period, one from the first year after surgery was chosen if possible. All CT examinations used for body composition analysis included a complete CT staging examination of the chest, abdomen, and pelvis. For each patient one preoperative and one postoperative CT dataset were selected for analysis. CT scans with insufficient image quality that might degrade body composition analysis were excluded.

### 2.3. CT Body Composition Analysis

**Figure 1:**
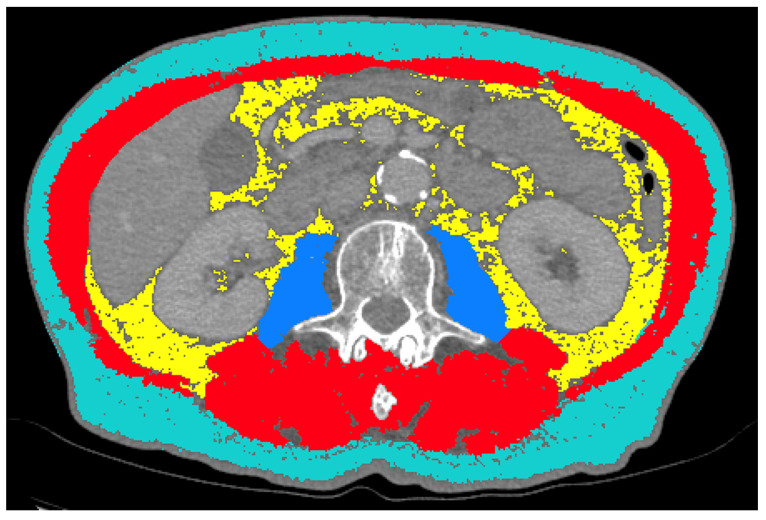
Example of semiautomated segmentations of subcutaneous adipose tissue (SAT; turquoise), visceral adipose tissue (VAT; yellow), psoas muscle area (PMA; blue) and total abdominal muscle area (TAMA, red + blue) at the L3 level.

DICOM (Digital Imaging and Communications in Medicine) datasets of each CT examination were extracted from the institutional PACS (Picture Archiving and Communication System), anonymized and transferred to the specific analysis tools. For analysis, the thinnest available slices were selected (ranging from 0.625 mm to 5 mm). A 2D segmentation was performed using the sliceOmatic semi-automatic segmentation tool (v5.0, Tomovision, Magog, QC, Canada). A representative, axial image of the abdomen at the mid-level of the L3 vertebra was identified in each patient and transferred to the workbench. Semiautomated segmentation of the following tissues was performed on single slice images: psoas muscle area (PMA), total abdominal muscle area (TAMA), visceral adipose tissue (VAT) and subcutaneous adipose tissue (SAT) (Figure 1).

**Figure 2:**
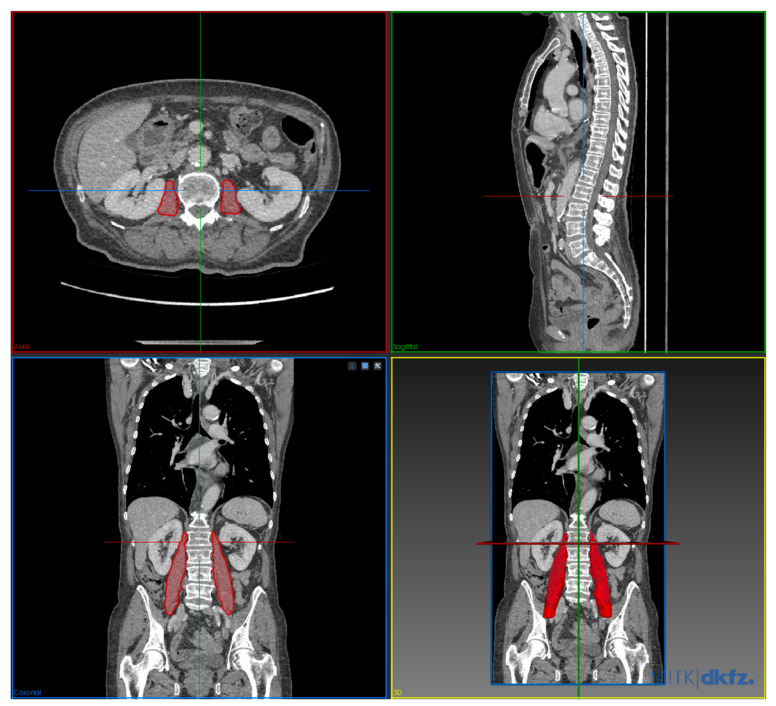
Example of manual 3D segmentation of psoas muscle volume in multiplanar reformation and 3D reconstruction.

Volumetry/3D segmentation of the psoas muscles was performed using the Medical Imaging Interaction Toolkit (MITK, German Cancer Research Center, Heidelberg, Germany). Separate segmentations for both psoas muscles were performed manually using the polygonal region of interest (ROI) tool. The segmentation is based on the planimetry method. Segmentation of the muscles was performed between the upper border of the L1 vertebra to the lower border of the S2 vertebra (Figure 2).

**Figure 3:**
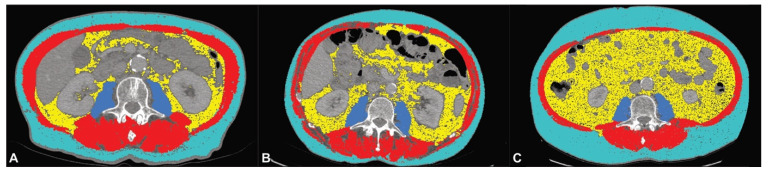
Example segmentations of patients without sarcopenia (**A**), sarcopenia (**B**) and sarcopenic obesity (**C**).

All segmentations were refined by a radiologist with >5 years of experience in abdominal imaging. Psoas muscle volume was normalized by the formula: (Volume _Right Psoas Muscle_ + Volume _Left Psoas Muscle_)/2. The abdominal adipose tissue ratio (ATR) was calculated by the formula: VAT/SAT. Relative changes in body composition parameters between the preoperative and postoperative follow-up scans were calculated by the formula: (Parameter_Follow-Up_ − Parameter_Preoperative_)/Parameter_Preoperative_ × 100. To determine whether a patient had sarcopenia, the lumbar skeletal muscle index at L3 (LSMI) was calculated by normalizing the TAMA by the patient’s height according to the formula: TAMA/body height ^2^. Patients with LSMI values ≤38.5 cm^2^/m^2^ for women and ≤52.4 cm^2^/m^2^ for men were classified as having sarcopenia, as previously published [26]. Sarcopenic obesity was defined as sarcopenia according to LSMI values in combination with evidence of obesity (BMI ≥ 30) [27] (Figure 3).

### 2.4. Statistical Analysis

Statistical analysis was performed using SPSS (version 25; IBM Corporation, Armonk, NY, USA) and Stata (version 17, StataCorp LLC, College Station, TX, USA). Normal distribution of the data was tested by Kolmogorov−Smirnoff test. According to the distribution of the variables, parametric or nonparametric tests were used for further analysis. Independent t-test (nonparametric: Mann−Whitney U-test) was used to identify statistical differences between the means of two groups and Pearson’s Chi-square test was used for categorical variables, respectively. Correlation analysis was performed using Pearson correlation (nonparametric: Spearman rank correlation). Uni- and multivariate regression analyses were performed to calculate odds ratios (OR) and to identify independent predictors. The Cox proportional-hazards model was used to investigate the association between the survival time of patients and the predictor variables. Results were considered statistically significant when *p* < 0.05.

## 3. Results

The patients’ characteristics are summarized in Table 1. Survival data (DFS and OS) was available for 76 patients (89.4%). There were no statistically significant correlations between sarcopenia or sarcopenic obesity and surgical technique (*p* = 0.631 and 0.958) or UICC stage (*p* = 0.631 and 0.961).

### 3.1. Analysis of Preoperative CT Body Composition Imaging Biomarkers

The results of the correlation analysis of the preoperative CT-body composition imaging biomarkers with postoperative complications are summarized in Table 2. The occurrence of complications was significantly higher in patients with higher SAT (*p* = 0.036). Binary univariate logistic regression showed a significantly increased risk of complications in patients with higher SAT (OR: 1.009, *p* = 0.027).

Major complications occurred more frequently in patients with sarcopenia (*p* = 0.045) and the occurrence of postoperative pneumonia showed a significant correlation with preoperative sarcopenic obesity (*p* = 0.019) (Table 3). Binary univariate logistic regression confirmed a significantly higher rate of major complications in sarcopenic patients (OR: 2.587, *p* = 0.048) and the higher risk of pneumonia in patients with sarcopenic obesity (OR: 6.364, *p* = 0.034).

**Figure 4:**
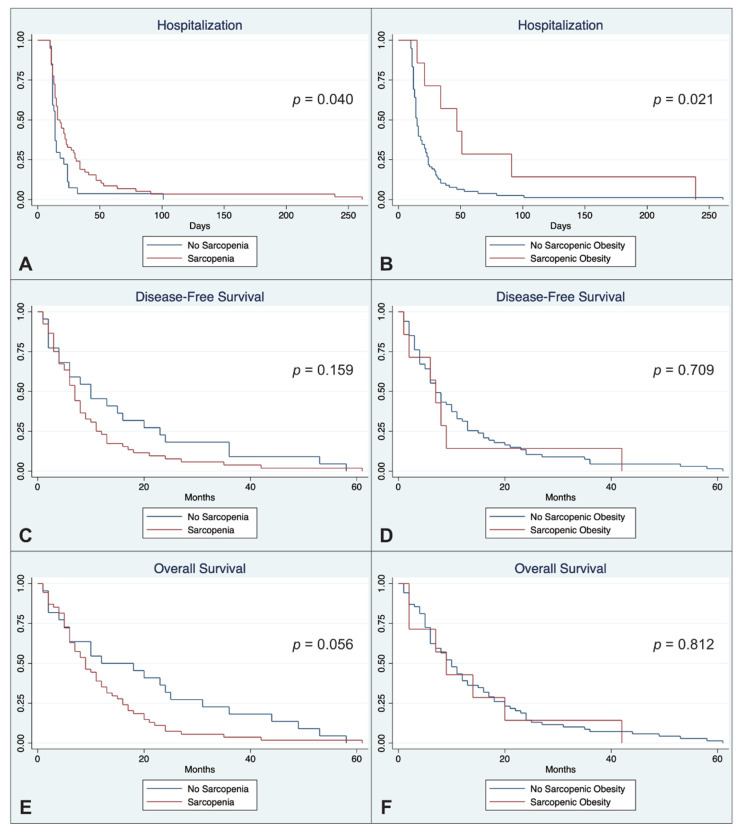
Kaplan-Meier duration of hospitalization (**A**,**B**), disease-free survival (**C**,**D**) and overall survival (**E**,**F**) stratified according to sarcopenia (left column) and sarcopenic obesity (right column).

Sarcopenic patients and patients with sarcopenic obesity were both at increased risk of prolonged postoperative hospitalization (31.9 d vs. 18.8 d and 71.1 d vs. 23.9 d, *p* = 0.040 and 0.021). Sarcopenia and sarcopenic obesity were also associated with shorter DFS and OS without statistical significance (*p* > 0.05). A statistical trend was shown for shorter OS in sarcopenic patients (12.1 vs. 20.0 months; *p* = 0.056) (Table 4 and Figure 4).

Univariate linear regression analysis showed a significant correlation between preoperative PMA (standardized beta coefficient: 0.795, *p* = 0.005), PMV (standardized beta coefficient: 0.055, *p* = 0.037) and OS. There were no significant correlations of duration of postoperative hospitalization, DFS and OS to the other preoperative body composition imaging biomarkers (VAT, SAT, TAMA, ATR, LSMI) (*p* > 0.05).

### 3.2. Analysis of Change in CT Body Composition Imaging Biomarkers during Follow-Up

Postoperative follow-up imaging was available for evaluation in 50 patients (59%). Relative changes in body composition imaging biomarkers in the postoperative follow-up scan compared to the preoperative CT examination were correlated with complications (Table 5) and postoperative outcome parameters. Patients with postoperative anastomotic leakage showed a significantly higher decrease in TAMA in the follow-up period than patients without anastomotic leakage (*p* = 0.032). Overall, patients with complications showed a (non-significant) higher decrease of muscle biomarkers than patients without complications (*p* > 0.05). DFS correlated significantly with ΔPMV (correlation coefficient: −0.387, *p* = 0.006) and ΔTAMA (correlation coefficient: −0.382, *p* = 0.007). Overall survival also showed a significant correlation with ΔPMV (correlation coefficient: −0.365 *p* = 0.009) and ΔTAMA (correlation coefficient: −0.356, *p* = 0.013). Multivariable linear regression analysis confirmed the significant correlation between shorter DFS and decrease in PMV (standardized beta coefficient: −0.308, *p* = 0.031) and TAMA (standardized beta coefficient: −0.316, *p* = 0.031) as well as the correlation between a shorter OS and decrease in PMV (standardized beta coefficient: −0.305, *p* = 0.035) and TAMA (standardized beta coefficient: −0.291, *p* = 0.044).

## 4. Discussion

The aim of this study was to assess the predictive value of body composition imaging biomarkers in CT on perioperative morbidity and survival after surgery in patients with locally advanced esophageal cancer. Our results are based on the largest cohort so far of surgically treated locally advanced EC and suggests that several important CT body composition imaging biomarkers can be used to predict peri- and postoperative morbidity and mortality. More prominent subcutaneous fat was associated with an increased risk of complications, and patients with preoperative sarcopenia had a higher risk of major complications after esophagectomy. Moreover, sarcopenic obesity was associated with a higher risk of postoperative pneumonia. Subsequently, patients with sarcopenia or sarcopenic obesity were at higher risk for prolonged postoperative hospitalization. Neither sarcopenia nor sarcopenic obesity was an independent risk factor for anastomotic leakage. A smaller preoperative abdominal muscle mass, identified by measurements of the psoas muscle, was associated with a shorter OS. Evaluation of postoperative follow-up imaging showed that patients with anastomotic leakage had a more marked decrease in muscle mass and that more pronounced muscle atrophy was associated with shorter DFS and OS. Our findings are consistent with previous studies demonstrating a poor overall outcome in patients with sarcopenia and sarcopenic obesity treated for different tumor entities, including upper GI cancer [28,29,30]. In addition, our results demonstrated that the decrease in muscle mass during postoperative follow-up may also indicate shorter DFS and OS. Our study extends available data on CT-body composition imaging biomarkers to patients with surgically treated locally advanced EC.

Patients with esophageal cancer have a decreased fat mass and higher rates of sarcopenia (about 50%) after neoadjuvant therapy [31]. All patients included in our study underwent neoadjuvant chemotherapy and had an even higher rate of sarcopenia (68.2%). The high incidence of sarcopenia was independent of the UICC tumor stage, so that the patient’s body composition did not allow reliable conclusions to be drawn about the preoperative tumor extent. Regarding adipose tissue, we found that patients with higher subcutaneous fat mass after neoadjuvant therapy had an elevated risk for surgical complications. The possible effects of neoadjuvant chemotherapy were not part of our current study; however, our results encourage further investigations regarding the effects of neoadjuvant therapy on patients’ body composition and the outcome of subsequent therapies. The role of sarcopenia in esophageal cancer treated with surgery and chemotherapy has already been investigated in smaller patient populations before. One study reported that sarcopenic patients were at a higher risk of developing a conduit necrosis [32]. Our results confirm the increased risk of complications for patients with sarcopenia in a larger population. However, our study revealed a generally increased risk for major complications in sarcopenic patients without a specific influence on the rate of anastomotic leakages. Besides the possible influence of sarcopenia on surgical morbidity, patient fitness seems to be an important factor in EC, as these patients have an impaired nutritional intake and digestion. Perioperative nutrition therapy has been proven to reduce postoperative risks and shorten intensive care duration in patients with EC [33]. Adipose and skeletal muscle tissues are considered as partly secretory organs, and VAT is associated with insulin resistance, which increases inflammatory cytokines levels. This might be a part of the pathophysiological explanation for prolonged wound healing and poorer outcome, especially in sarcopenic obesity [34,35] Our data suggest that patients with sarcopenic obesity are at a higher risk for postoperative pneumonia. This seems plausible, as obesity is often linked with respiratory problems. Prolonged recumbency also increases the risk of aspiration and sarcopenia might include a loss of respiratory muscle, leading to more difficult respiration in sarcopenic obese patients [36,37].

As the population is aging and comorbidities rise, sarcopenia is likely to become more prevalent in the future and might play an even more important role in patient care. There have been several attempts to establish risk scores for surgical mortality in esophageal cancer [38,39,40]. Future endeavors at calculating risk scores should consider taking CT body composition imaging biomarkers into account, especially because the preoperative CT is a standard procedure in the staging process. CT-based parameters enable a detailed analysis of an individual patient’s body composition. Imaging-based assessment could complement established conventional measures such as BMI or waist-to-hip-ratio, or even replace them. It is important to assess the individual patient’s fitness as the proportion of complex, extensive operations is becoming more frequent as surgical techniques improve. Therefore, one needs to weigh the benefits of a potentially curative operation against its potentially high morbidity to evaluate alternative treatment strategies in vulnerable patients [41]. When patients at a higher risk are identified before surgery, specific interventions such as improving nutritional intake and/or assisted physical activity might be incorporated into the peri- and postoperative management [32,42]. A major drawback of the CT body composition approach as used in this study is that it is time-consuming, which may hinder its routine clinical use. However, our results could pave the way to automated, standardized analysis.

Our study has some limitations. The major limitation is its single-center, retrospective design, which means that the results may not be generalizable and should be confirmed in a prospective trial and compared to conventional parameters such as body impedance analysis or muscle function. The study design prohibits any valid conclusion to be drawn regarding potential causal relationships of the tumor burden, daily food intake and physical activities with the manifestation of sarcopenia. Nonstandardized imaging intervals could have biased the body composition results in our patients. Even though the cohort is comparatively large, more data are needed to determine the thresholds of these imaging biomarkers.

## 5. Conclusions

In conclusion, our study highlights the predictive value of CT body composition imaging biomarkers to identify high-risk patients with locally advanced esophageal cancer undergoing surgery after neoadjuvant treatment. Sarcopenic patients are at an increased risk for major complications, and patients with sarcopenic obesity are more prone to postoperative pneumonia. Sarcopenia and sarcopenic obesity are both subsequently associated with a prolonged postoperative hospitalization. Low preoperative muscle mass and its decrease during postoperative follow-up are associated with shorter disease-free and overall survival.

## Figures and Tables

**Table 1:** Patient characteristics.

Characteristic	Factor	Number (%)/Mean (SD)	Range
Sex	Female	10 (11.8%)	
	Male	75 (88.2%)	
Age (years)		64.3 (9.8)	45–83
BMI		26.79 (4.01)	16–40
Surgical technique	Open	8 (9.4%)	
	Laparoscopic	51 (60%)	
	Hybrid	13 (15.3%)	
	Robotic	13 (15.3%)	
UICC stage (missing information in *n* = 3 patients)	I	7 (8.2%)	
	II	18 (21.2%)	
	III	49 (57.6%)	
	IV	8 (9.4%)	
Neoadjuvant chemotherapy	No	0 (0.0%)	
	Yes	85 (100.0%)	
Neoadjuvant chemoradiotherapy	No	67 (78.8%)	
	Yes	18 (21.2%)	
Complications (overall)	No	24 (28.2%)	
	Yes	61 (71.8%)	
Major complications	No	40 (47.1%)	
	Yes	45 (52.9%)	
Anastomotic leakage	No	74 (87.1%)	
	Yes	11 (12.9%)	
Pneumonia	No	58 (68.2%)	
	Yes	27 (31.8%)	
Hospitalization (d)		27.75 (38.75)	10–261
DFS (months)		11.51 (13.01)	0–61
OS		14.38 (13.75)	1–61
VAT (cm^2^)		156.37 (89.95)	6.62–399.70
SAT (cm^2^)		166.25 (71.59)	14.00–393.30
ATR		0.977 (0.516)	0.04–2.44
PMA (cm^2^)		19.89 (5.50)	9.92–37.96
PMV		183.75 (59.87)	67.04–346.91
TAMA (cm^2^)		147.98 (30.26)	78.40–228.13
LSMI (cm^2^/m^2^)		47.44 (7.91)	30.63–68.42
Sarcopenia	No	27 (31.8%)	
	Yes	58 (68.2%)	
Sarcopenic obesity	No	78 (91.8%)	
	Yes	7 (8.2%)	

**Table 2:** Correlation analysis of preoperative CT body composition imaging biomarkers to postoperative complications.

**Outcome**		**Preoperative CT Body Composition Imaging Biomarkers**							
		**Adipose Tissue**							
		**VAT**	***p***	**SAT**	***p***	**ATR**	***p***		
		**Mean (SD)**		**Mean (SD)**		**Mean (SD)**			
Complications	Yes	156.77 (90.63)	0.946	177.30 (73.43)	0.036	0.90 (0.47)	0.056		
	No	155.38 (90.12)		138.17 (59.20)		1.17 (0.59)			
Major complications	Yes	158.45 (90.70)	0.850	180.02 (75.27)	0.108	0.89 (0.44)	0.149		
	No	154.04 (90.20)		150.76 (64.67)		1.08 (0.58)			
Anastomotic leakage	Yes	168.01 (68.71)	0.596	183.70 (85.31)	0.647	0.97 (0.33)	0.793		
	No	154.64 (92.96)		163.65 (69.62)		0.98 (0.54)			
Pneumonia	Yes	164.53 (90.19)	0.604	190.24 (84.96)	0.065	0.92 (0.46)	0.491		
	No	152.58 (90.38)		155.08 (62.13)		1.00 (0.54)			
		**Muscle tissue**							
		**PMA**	***p***	**PMV**	**p**	**TAMA**	***p***	**LSMI**	***p***
		**Mean (SD)**		**Mean (SD)**		**Mean (SD)**		**Mean (SD)**	
Complications	Yes	20.01 (5.55)	0.653	177.39 (58.04)	0.101	146.90 (27.65)	0.911	47.38 (7.36)	0.762
	No	19.57 (5.48)		199.92 (62.64)		150.73 (36.59)		47.59 (9.35)	
Major Complications	Yes	19.46 (5.82)	0.235	182.29 (59.50)	0.460	146.59 (25.32)	0.843	47.05 (5.93)	1.000
	No	20.36 (5.15)		185.43 (61.01)		149.54 (35.28)		47.88 (9.74)	
Anastomotic leakage	Yes	21.17 (7.59)	0.804	199.08 (75.06)	0.591	151.22 (26.72)	0.778	48.21 (6.63)	0.530
	No	19.70 (5.16)		181.47 (57.55)		147.50 (30.89)		47.32 (8.12)	
Pneumonia	Yes	20.06 (6.62)	0.981	178.41 (64.53)	0.565	140.91 (28.42)	0.164	45.48 (6.40)	0.199
	No	19.81 (4.96)		186.24 (58.00)		151.27 (30.77)		48.35 (8.42)	

VAT = visceral adipose tissue; SAT = subcutaneous adipose tissue; ATR = adipose tissue ratio, PMA = psoas muscle area; PMV = psoas muscle volume; TAMA = total abdominal muscle area, LSMI = lumbar skeletal muscle index.

**Table 3:** Correlation analysis of sarcopenia and sarcopenic obesity in relation to postoperative complications.

Outcome		Sarcopenia			Sarcopenic Obesity		
		Yes	No	*p*	Yes	No	*p*
Complications	Yes	45	16	0.081	7	54	0.083
	No	13	11		0	24	
Major Complications	Yes	35	10	0.045	6	39	0.070
	No	23	17		1	39	
Anastomotic leakage	Yes	8	3	0.732	2	9	0.198
	No	50	24		5	69	
Pneumonia	Yes	22	5	0.073	5	22	0.019
	No	36	22		2	56	

**Table 4:** Correlation analysis of sarcopenia and sarcopenic obesity to postoperative outcome parameters.

Outcome		Hazard Ratio	95% CI		*p*
			Lower	Upper	
Hospitalization (d)	Sarcopenia	0.611	0.382	0.977	0.040
	Sarcopenic Obesity	0.394	0.179	0.870	0.021
DFS (months)	Sarcopenia	1.444	0.866	2.406	0.159
	Sarcopenic Obesity	1.162	0.529	2.550	0.709
OS (months)	Sarcopenia	1.656	0.987	2.781	0.056
	Sarcopenic Obesity	1.099	0.503	2.402	0.812

DFS = disease-free survival; OS = overall survival.

**Table 5:** Correlation analysis of relative changes in CT body composition imaging biomarkers to postoperative complications.

**Outcome**		**Relative Changes in CT Body Composition Imaging Biomarkers between Pre- and Postoperative Scans**							
		**Adipose tissue**							
		**ΔVAT**	***p***	**ΔSAT**	***p***	**ΔATR**	***p***		
		**Mean (SD)**		**Mean (SD)**		**Mean (SD)**			
Complications	Yes	−48.96 (32.39)	0.905	−31.62 (28.17)	0.234	−34.31 (23.47)	0.849		
	No	−50.63 (22.07)		−22.14 (24.26)		−30.71 (35.93)			
Major Complications	Yes	−49.92 (32.68)	0.544	−30.53 (29.56)	0.477	−34.22 (30.01)	0.802		
	No	−48.65 (26.42)		−27.40 (24.51)		−28.13 (37.15)			
Anastomotic leakage	Yes	−57.41 (41.51)	0.268	−32.91 (40.58)	0.860	−48.54 (41.66)	0.069		
	No	−47.74 (27.32)		−28.44 (24.38)		−28.16 (30.42)			
Pneumonia	Yes	−46.44 (36.37)	0.681	−37.81 (25.74)	0.281	−22.53 (47.17)	0.322		
	No	−50.77 (26.85)		−25.16 (27.44)		−35.89 (23.48)			
		**Muscle tissue**							
		**ΔPMA**	***p***	**ΔPMV**	***p***	**ΔTAMA**	***p***	**ΔLSMI**	***p***
		**Mean (SD)**		**Mean (SD)**		**Mean (SD)**		**Mean (SD)**	
Complications	Yes	−8.08 (16.15)	0.063	−8.03 (23.74)	0.803	−7.63 (10.13)	0.105	−63.29 (10.38)	0.886
	No	1.20 (14.29)		−7.19 (14.61)		−1.59 (10.85)		−37.24 (99.82)	
Major Complications	Yes	−6.68 (15.52)	0.369	−10.39 (23.36)	0.552	−8.17 (10.61)	0.168	−63.60 (11.10)	0.490
	No	−4.40 (17.12)		−4.00 (19.06)		−3.29 (10.03)		−47.24 (77.14)	
Anastomotic leakage	Yes	−14.78 (18.76)	0.183	−20.04 (38.23)	0.568	−15.04 (12.09)	0.032	−61.50 (19.44)	0.319
	No	−3.85 (15.07)		−5.15 (15.72)		−4.26 (9.34)		−55.64 (55.36)	
Pneumonia	Yes	−12.79 (20.45)	0.145	−2.72 (24.04)	0.362	−9.13 (9.43)	0.158	−62.52 (13.75)	0.747
	No	−2.39 (12.61)		−10.46 (20.38)		−4.67 (10.87)		−53.88 (61.15)	

VAT = visceral adipose tissue; SAT = subcutaneous adipose tissue; ATR = adipose tissue ratio, PMA = psoas muscle area; PMV = psoas muscle volume; TAMA = total abdominal muscle area, LSMI = lumbar skeletal muscle index.

## Data Availability

The data presented in this study are available on request from the corresponding author.
